# Morphological and Histopathological Changes in Orofacial Structures of Experimentally Developed Acromegaly-Like Rats: An Overview

**DOI:** 10.1155/2012/254367

**Published:** 2012-02-28

**Authors:** Masahiro Iikubo, Ikuho Kojima, Maya Sakamoto, Akane Kobayashi, Hidetoshi Ikeda, Takashi Sasano

**Affiliations:** ^1^Department of Oral Diagnosis, Tohoku University Graduate School of Dentistry, 4-1 Seiryo-machi, Aoba-ku, Sendai, Miyagi 980-8575, Japan; ^2^Research Institute for Pituitary Disease, Southern Tohoku General Hospital, 7-115, Yatsuyamada, Koriyama, Fukushima 963-8563, Japan

## Abstract

Tongue enlargement and mandibular prognathism are clinically recognized in almost all patients with acromegaly. An acromegaly-like rat model recently developed by exogenous administration of insulin-like growth factor I (IGF-I) was used to investigate morphological and histopathological changes in orofacial structures and to clarify whether these changes were reversible. Exogenous administration of IGF-I evoked specific enlargement of the tongue with identifiable histopathological changes (increased muscle bundle width, increased space between muscle bundles, and increased epithelial thickness), elongation of the mandibular alveolar bone and ascending ramus, and lateral expansion of the mandibular dental arch. Regarding histopathological changes in the mandibular condyle, the cartilaginous layer width, bone matrix ratio, and number of osteoblasts were all significantly greater in this rat model. After normalization of the circulating IGF-I level, tongue enlargement and histopathological changes in the tongue and mandibular condyle were reversible, whereas morphological skeletal changes in the mandible remained.

## 1. Introduction

In acromegalic or acrogiantic patients, growth hormone (GH) is oversecreted, generally due to a GH-secreting pituitary adenoma (GH-oma). GH stimulates the production of insulin-like growth factor I (IGF-I), which mediates the growth-promoting actions of GH. IGF-I overexpression causes a variety of gradual morphological orofacial changes, such as tongue enlargement, lip thickening, increased sinus volume, and mandibular prognathism [1–6]. These changes are considered the most definitive signs of acromegaly (acrogiantism) and are found in almost all cases. Enlargement of the tongue and mandibular protrusion often cause masticatory disturbances, dysphagia, speech difficulties, and/or aesthetic problems, resulting in a marked decrease in the patient's quality of life. When devising a treatment plan for these functional disturbances, it is important to know whether these enlarged tissues improve after IGF-I serum levels return to normal. However, there are no detailed reports regarding the morphological or histopathological changes in orofacial structures that accompany acromegaly, probably because of difficulty of the evaluation in human subjects. Consequently, whether such changes are reversible after normalization of the circulating IGF-I levels in humans following removal of a GH-oma is unknown.

This paper will focus on the time course of tongue enlargement and jaw bone growth during IGF-I overexpression and after IGF-I normalization, based on a series of studies using an acromegaly-like rat model developed by exogenous administration of IGF-I [7–10].

## 2. Animal Preparation

Prior approval for all research protocols was obtained from the Institute for Experimental Animals at Tohoku University School [7–10], the experimental animals were male Wistar rats at 10 weeks of age [7–10]. During the 8-week experimental period, animals were housed individually in a controlled environment at 23°C with a 12 h light/dark cycle and with free access to tap water and food. The rats were divided into two equal groups: an experimental group treated with human recombinant IGF-I and a sham control group. Human recombinant IGF-I (SOMAZON) was kindly provided by Astellas Pharma, Inc. (Tokyo, Japan). For 4 weeks, rats in the IGF-I group were continuously subcutaneously infused with 640 *μ*g/day IGF-I dissolved in saline using implanted osmotic minipumps (Model 2002, Alza Corp., Palo Alto, CA, USA). Rats in the control group were infused with saline alone by the same method. After the pumps had been removed at 14 weeks of age, rats were housed (as described) for an additional 4 weeks. All surgical procedures for the implantation and removal of pumps were performed under ether anesthesia. 

## 3. Effects of IGF-I Administration

### 3.1. IGF-I Serum Level and Body Weight

**Figure 1:**
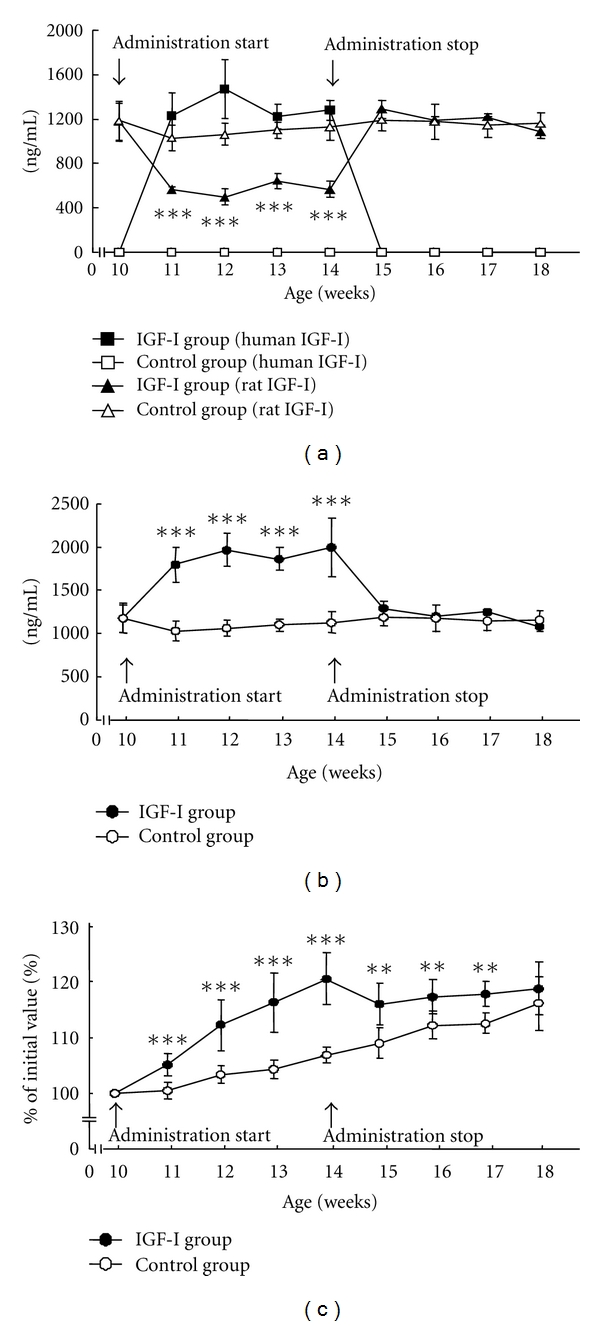
(a, b) Serum levels of human and rat IGF-I (a), and total IGF-I (b) during and after administration of human IGF-I (***P < 0.001 versus control). (c) Body weight, as a percentage of initial value in the same animal, during and after administration of human IGF-I (**P < 0.01, ***P < 0.001 versus control). In (a)–(c), data represent the mean ± SD of six rats, adapted from a previous report [7].

Serum levels of rat IGF-I and human IGF-I were measured by radioimmunoassay [7, 11]. As show in Figure 1(a), human IGF-I levels in the serum increased immediately and remained at a high level throughout the administration period [7]. In contrast, the level of endogenous rat IGF-I decreased significantly during the administration period [7], indicating that some negative feedback mechanism(s) may be involved. Similarly, Bermann et al. [12] previously reported that recombinant human IGF-I exerts a powerful negative feedback on GH secretion in healthy humans, stimulating hypothalamic somatostatin secretion in particular. Thus, it seems likely that the circulating level of endogenous rat IGF-I decreased due to suppression of the rat's own GH concentration. In the rat model, the total circulating IGF-I (human + rat) was approximately 1.7 to 1.9 times higher than in the control group (Figure 1(b)). Body weight increased during IGF-I administration (Figure 1(c)) [7].

### 3.2. Tongue

**Figure 2:**
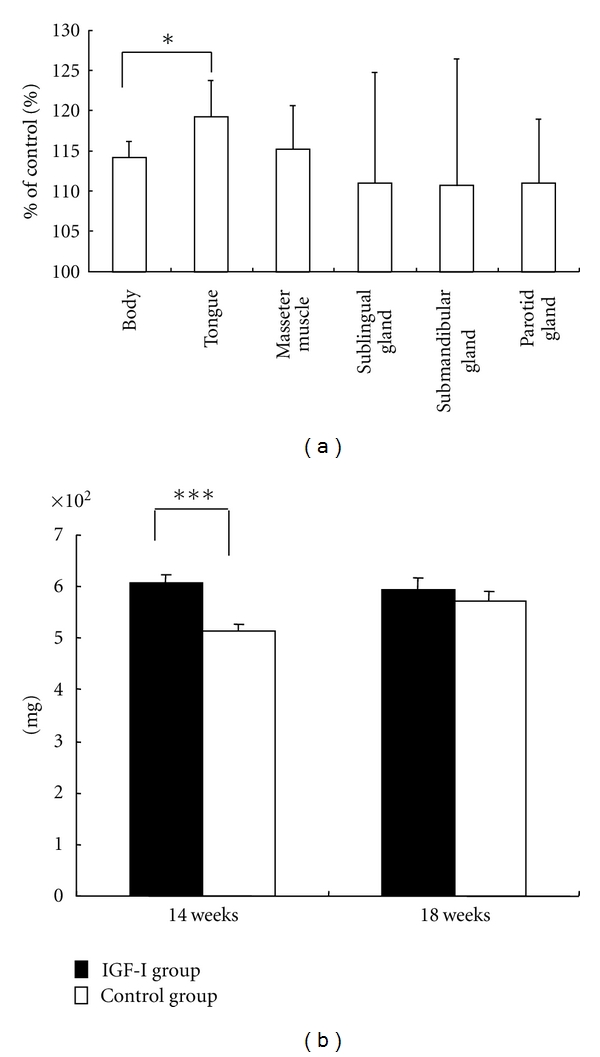
(a) Changes in body weight and soft oral tissue weight at 14 weeks of age in the IGF-I group compared to control (*P < 0.05 versus total body weight). (b) Tongue weight at 14 and 18 weeks of age in IGF-I-treated and control rats (***P < 0.001 versus control). Data represent the mean ± SD of six rats, adapted from a previous report [8].

**Figure 3:**
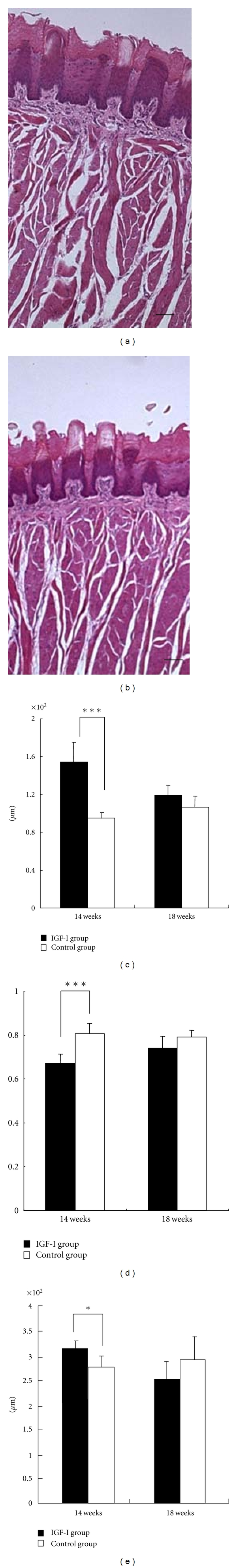
(a, b) Coronal sections of the dorsal superior longitudinal tongue muscle from an IGF-I-treated rat (a) and a control rat (b) at 14 weeks of age (hematoxylin-eosin staining, 100x magnification, bars = 100 *μ*m). (c–e) Muscle bundle width (c), ratio of the muscle parts (d), and total epithelial thickness (e). In (c–e), data represent the mean ± SD of six rats (*P < 0.05, **P < 0.01, ***P < 0.001 versus control); adapted from a previous report [8].

In the rat model, enlargement of the tongue was observed after 4 weeks of IGF-I treatment and was specific to the tongue compared to other oral soft tissues (i.e., masseter muscle, sublingual gland, submandibular gland, and parotid gland) (Figures 2(a) and 2(b)) [8]. This is consistent with clinical reports showing the occurrence of an enlarged tongue to be substantial (from 84% to 95%) in patients with acromegaly [1, 2]. We conducted histopathological examination of specimens stained with hematoxylin-eosin to analyze factors that lead to tongue enlargement [8] (Figures 3(a) and 3(b)). No demonstrable change in the subepithelial connective tissue could be seen in our rat model (Figures 3(a) and 3(b)), although one previous clinical case report did show increased subepithelial connective tissue [13]. Muscle bundle width and thickness of the epithelium were both significantly greater in the rat model than in control rats [8] (Figures 3(c) and 3(e)). This result is supported by a clinical report showing that acromegalic tongues at autopsy had enlarged lingual muscle fibers (twice as large as in normal subjects) and a thickened tongue epithelium due to marked acanthosis [13]. Desnoyers et al. [14] have shown that the IGF receptor is distributed in myofibrils of the rat tongue, and Donath et al. [15] demonstrated that IGF-I stimulates expression of myofibrillar gene. Taking these observations together, it seems clear that IGF-I stimulated the proliferation of myofibrils in our rat model. In addition, the ratio of muscle parts in the rat model was significantly smaller than in the control (Figure 3(d)), indicating that the spaces between muscle bundles in the rat model increased [8]. Increased spaces between muscle bundles may indicate, at least in part, an increase in extracellular fluid component. This also agrees well with clinical reports in humans [16–20], which indicate that increases in extracellular fluid volume in acromegaly are caused by the active efflux of sodium from the intracellular pool due to activation of Na^+^-K^+^ transport, and that skeletal muscle potassium content is higher in patients with acromegaly than in weight-matched healthy controls. Furthermore, Dorup and clausen [20] demonstrated that IGF-I stimulates the Na^+^-K^+^ pump and increases ^42^K uptake and ^22^Na efflux in isolated rat soleus muscle.

### 3.3. Jaw Bones

**Figure 4:**
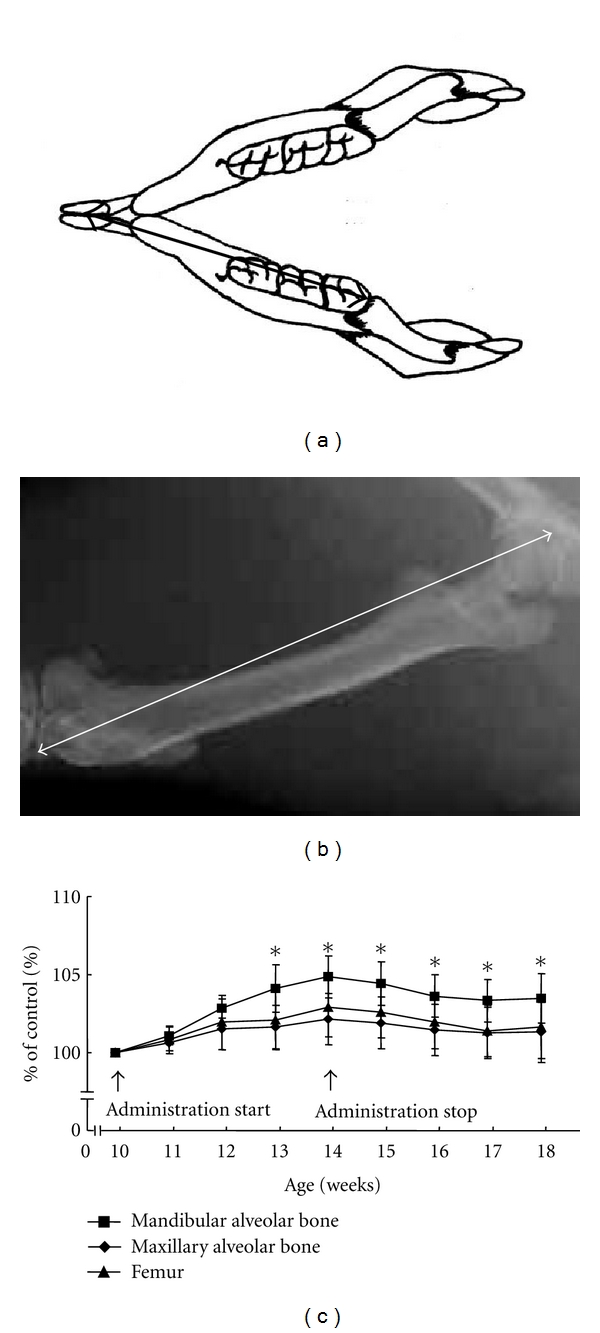
(a, b) Illustration showing length measurements of the mandibular alveolar bone (a) and femur (b). Measurement of the maxillary alveolar bone (not shown) was the same as for the mandible. (c) Bone length during and after administration of human IGF-I, shown as a percentage of bone length in the control group at the same time (*P < 0.05, mandibular alveolar bone versus other bones). Data represent the mean ± SD of six rats, adapted from a previous report [7].

We compared the enlargement of the mandible with that of the maxilla and femur in the rat model to verify clinical findings showing the enlargement of the mandible as the most severe skeletal change in acromegalic patients [2, 3, 7, 21]. The length of the alveolar bones of the mandible and maxilla were evaluated on a plaster model obtained from a precise impression of each dental arch (Figure 4(a)). The lengths of the right femurs were measured on X-ray photographs (Figure 4(b)). The mandibular alveolar bone, maxillary alveolar bone, and femur were all elongated during IGF-I administration (Figure 4(c)) [7]. Elongation was greatest in the mandible, indicating that the amount of bone growth differed from site to site. Malpe et al. [22] have reported that levels of IGFs and IGF-binding proteins (IGFBP-1 to -6), which have inhibitory or stimulatory modulating effects on the local actions of IGF, varied significantly among skeletal sites (calvaria, mandible, rib, vertebra, and marrow stroma) in humans. Furthermore, they reported that human calvarial and mandibular cells produce especially high levels of IGFBP-5, which stimulates bone cell proliferation and potentiates the actions of IGFs. These site-specific IGFBP levels suggest a possible mechanism for our finding that the growth rate was higher in the mandible than in either the maxilla or femur. Over a century ago, Chalk [23] and Benda [24] concluded that an enlarged tongue may be related to the etiology of the mandibular prognathism seen in patients with acromegaly. Clinical findings also suggest that an enlarged tongue affects the size and shape of the jaw [1].

**Figure 5:**
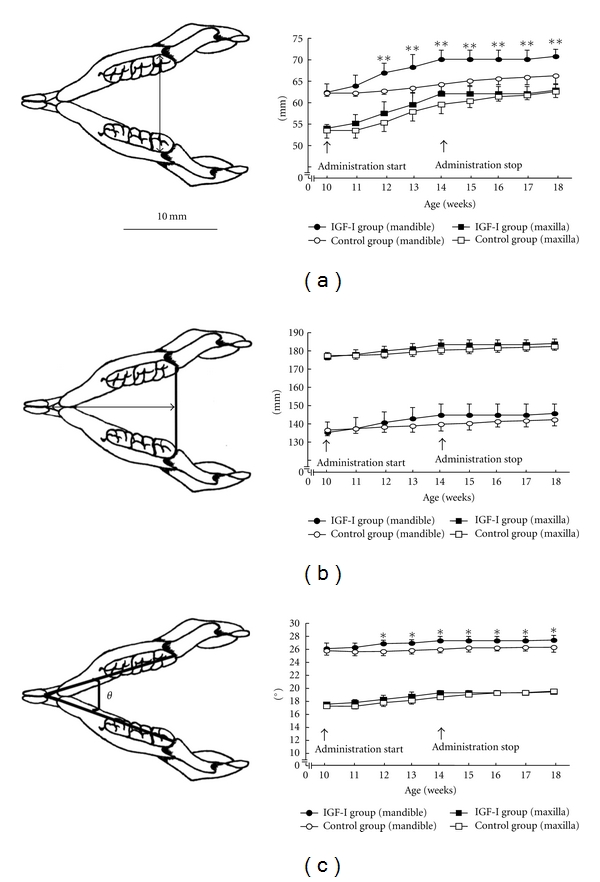
Left: illustration showing measurements for width (a), length (b), and angle *θ* (c) of the mandibular dental arch. Measurements for the maxillary arch (not shown) were the same as for the mandible. Right: mandibular and maxillary arch measurements during and after administration of IGF-I (mandible: *P < 0.05, **P < 0.01 versus control; maxilla: no significant differences). Closed circles: IGF-I-treated mandibles; open circles: control mandibles; closed squares: IGF-I-treated maxilla; open squares: control maxilla. Data represent the mean ± SD of six rats; adapted from a previous report [9].

To clearly detail the changes that occur in the dental arches of patients with acromegaly, we investigated the time course of changes in the width, length, and angle of the mandibular and maxillary dental arches in rat models using the plaster models mentioned above (Figures 5(a)–5(c)) [9]. We found that mandibular arch growth was greater in the lateral direction than in the anteroposterior direction (Figures 5(a)–5(c)) [9]. This result is consistent with a clinical report showing increased lateral growth in the mandibles of humans with acromegaly, which is expected to induce the lateral cross-bite in these patients [25]. Indeed, a previous study using plaster models of 28 case of acromegaly demonstrated that the mandible was larger than the maxilla in 32% of patients, and that the maxilla was captured in the mandible in one patient [5]. An enlarged tongue should be one of the explanation of the remarkable lateral dental arch expansion in the mandible because tongue enlargement would have the potential to widen the arch by applying lateral pressure to the teeth.

**Figure 6:**
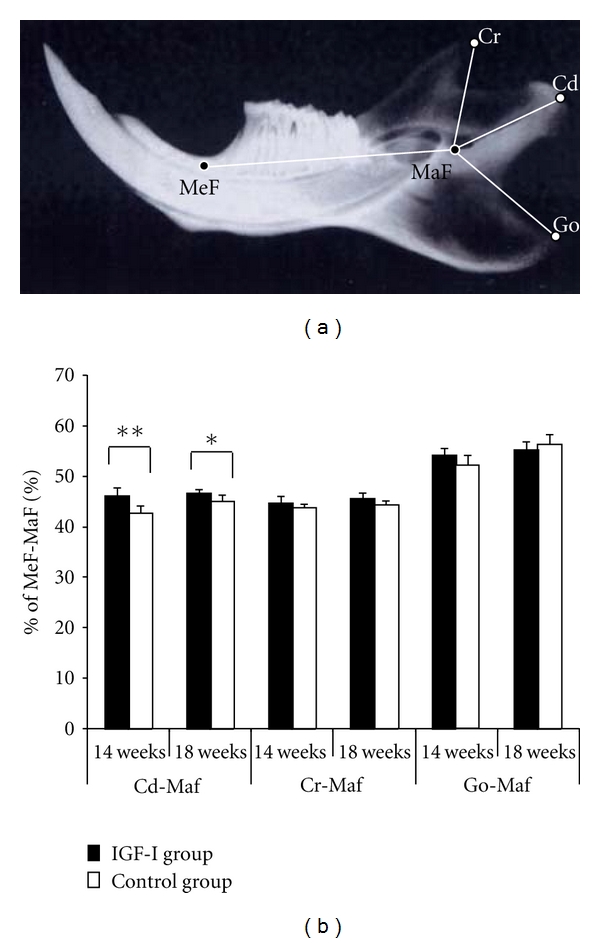
(a) Lateral cephalometric image showing the basal points for mandibular morphological analysis. MaF: most inferior point of the mandibular foramen; MeF: mental foramen; Cd: most posterior point of the condyle; Cr: tip of the coronoid process; Go: tip of the angular process. (b) Mandibular morphological change as a percentage of MeF-MaF in the same animal. Data represent the mean ± SD of six rats (*P < 0.05, **P < 0.01 versus control), adapted from a previous report [10].

**Figure 7:**
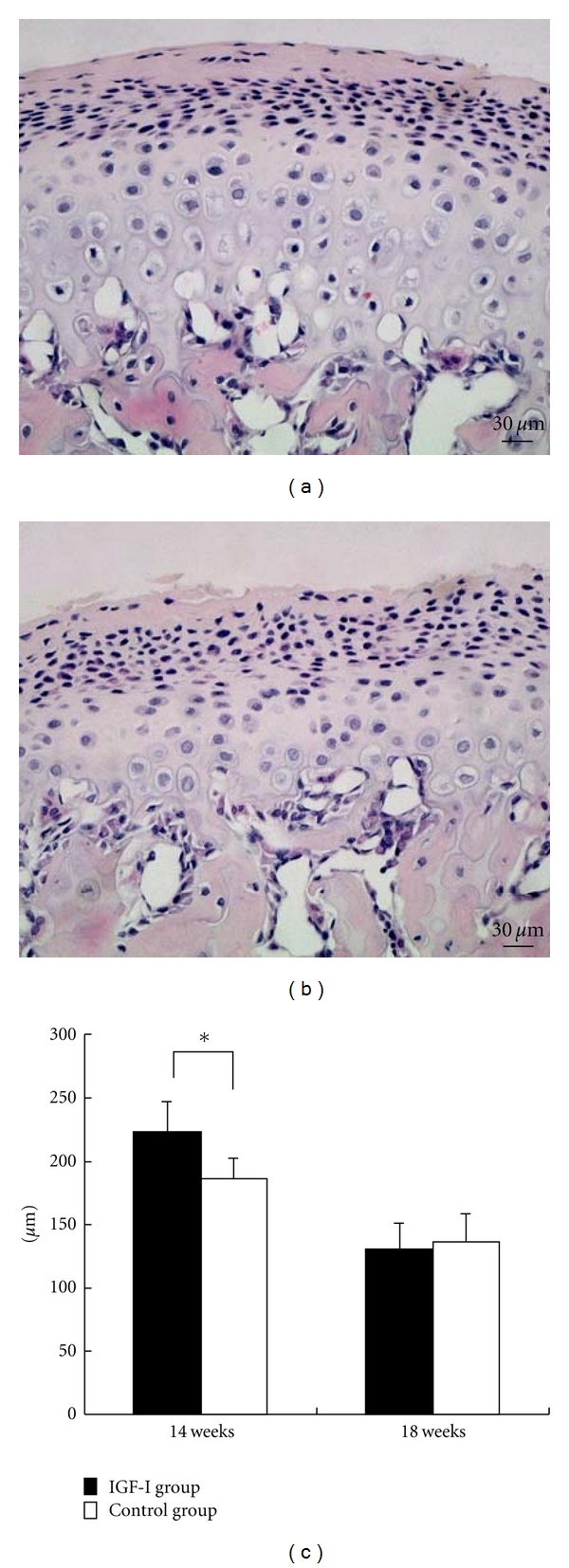
(a, b) Coronal sections of the cartilaginous layer in the mandibular condyle in the IGF-I group (a) and the control group (b) at 14 weeks of age (hematoxylin-eosin staining, 200x magnification, bars = 30 *μ*m). (c) Width of the cartilaginous layer. Data represent the mean ± SD of six rats (*P < 0.05 versus control); adapted from a previous report [10].

Kunzler and Farmand [2] observed 31 patients with persistent symptoms of GH-oma. Using X-ray photographs of the facial skeleton, they reported that these patients exhibited significant bone changes only in the mandible (length of the ascending ramus, length of the mandibular body, and chin prominence). In our rat model, the length of the ascending ramus was specifically and significantly increased, but there were no significant increases in the length of the coronoid or angular processes (Figures 6(a) and 6(b)) [10]. These findings are supported by published evidence that IGF-I stimulates chondrocyte activity in the growth plate and promotes bone elongation [26–28], and that normal growth in the ascending ramus is due to endochondral ossification of the mandibular condyle [29–31]. In the rat model, histopathological changes in the mandibular condyle show a marked increase in width of the cartilaginous layer (Figures 7(a)–7(c)) [10]. Regarding the effect of IGF-I on the articular cartilage of the mandibular condyle, Blumenfeld et al. [32] reported that *in vitro* supplementation of IGF-I to cultured mandibular condyle explants induced an increase in the cartilaginous layer width in mice. Suzuki et al. [33] also reported that local administration of IGF-I to articular capsules of the mandibular condyle increased the amount of endochondral ossification in adult rats. In addition, immunohistological examination of rat mandibular condyle shows that IGF-I receptors exist mainly in the condylar cartilage [34]. It therefore seems indisputable that IGF-I stimulates endochondral ossification in the mandibular condyles of the rat models. Furthermore, Dellatte et al. [35] reported that cartilaginous growth in the mandibular condyle appears to be more sensitive to IGF-I than that in the femoral head, presumably due to their different cartilage origins (primary and secondary cartilage). Specific sensitivity to IGF-I in the mandibular condyle may explain the specific mandibular protrusion seen in acromegalic patients.

**Figure 8:**
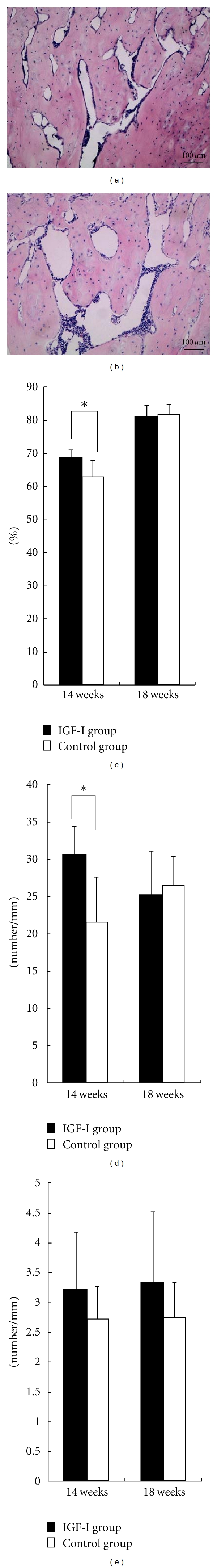
(a, b) Coronal sections of bone matrix in the mandibular condyle in the IGF-I group (a) and the control group (b) at 14 weeks of age (hematoxylin-eosin staining, 100x magnification, bars = 100 *μ*m). (c–e) Bone matrix ratio (c), number of osteoblasts (d), and number of osteoclasts (e). Data represent the mean ± SD of six rats (*P < 0.05 versus control), adapted from a previous report [10].

To further clarify the bone formation changes that occur in the mandibular condyle, we also investigated the bone matrix ratio (the ratio of bone matrix area in the bone tissue area) and the numbers of osteoblasts and osteoclasts [10]. The bone matrix ratio was measured on specimens stained with hematoxylin-eosin (Figures 8(a) and 8(b)). Osteoblasts and osteoclasts were identified by osteocalcin immunoreactivity and by detection of tartrate-resistant acid phosphatase (TRAP), respectively, with Carazzi's hematoxylin for counterstaining [10]. The bone matrix ratio and number of osteoblasts in the mandibular condyle were significantly greater in rat model compared to control rats (Figures 8(a)–8(e)) [10]. This indicates that administration of IGF-I promotes proliferation of osteoblasts, thereby accelerating bone formation in that part of the mandibular condyle. In agreement with our results, Mueller et al. [36] and Wakisaka et al. [37] reported that administration of IGF-I increases the number of osteoblasts, the trabecular bone mass, and the bone formation rate. It is now clear that IGF-I stimulates osteoblasts and promotes bone formation. There are, however, discrepancies in researchers' reports on osteoclasts and IGF-I administration. One publication reported that IGF-I induces a decrease in the number of osteoclasts in rat femur and tibia [38], a second reported an increase in mouse calvarial bone [39], and a third reported no change in rat femur [37]. Our findings showed no significant intergroup differences in the number of osteoclasts, indicating that IGF-I stimulates osteoclasts less than it does osteoblasts (Figures 8(d) and 8(e)) [10].

## 4. Changes after Cessation of IGF-I Administration

### 4.1. IGF-I Serum Level and Body Weight

After IGF-I administration was stopped, human IGF-I levels immediately decreased to zero, and rat IGF-I levels immediately returned to levels seen in the control group, so that total serum IGF-I levels were similar in the two groups (Figures 1(a) and 1(b)) [7]. This may be considered consistent with our clinical finding that the serum IGF-I level normalizes after successful transsphenoidal surgery in patients with a GH-oma [40]. In two other animal models of acromegaly or acromegaly-like disease (osteocalcin-IGF-I transgenic mice [41] and GH transgenic mice [42]), investigators were unable to assess morphological changes after IGF-I normalization because serum levels of IGF-I in these animal models never returned to normal. Thus, our rat model appears to offer the advantage of allowing us to assess how changes occur both during the IGF-I administration itself and after normalization of the circulating IGF-I level (incidental to discontinuation of the administration). Body weight fell somewhat immediately after administration ended and finally settled at the same level as the control group (Figure 1(c)) [7]. These data agree well with a clinical report [43] showing that the body weight of patients with a GH-oma decreases rapidly for 2 weeks after successful treatment (in line with rapid decreases in total body water and body cell mass).

### 4.2. Tongue

No intergroup differences in tongue weight were detected between the IGF-I and control groups after IGF-I administration ended (Figure 2(b)) [8]. Similarly, neither muscle bundle width, ratio of the muscle parts, nor thickness of the epithelium showed any significant differences between the IGF-I and control groups after administration ended (Figures 3(c)–3(e)) [8]. Thus, changes in the tongue after overexpression of IGF-I were reversible, suggesting that enlargement of the tongue in patients with acromegaly should improve after normalization of the circulating IGF-I level. Several clinical reports have described changes in skeletal muscle after successful treatment (surgery) in patients with acromegaly. Decreases in skeletal muscle volume (observed by multiscan computed tomography [44]) and skeletal muscle potassium content (due to decreased stimulation of the Na^+^-K^+^ pump [19]) agree well with our findings. The decrease in the space between muscle bundles in our rat model may be related to decreased stimulation of the Na^+^-K^+^ pump caused by normalization of circulating IGF-I levels.

### 4.3. Jaw Bones

Disharmonious jaw growth, due to excessive mandibular alveolar bone enlargement and elongation of condylar length, persisted after normalization of the circulating IGF-I level (Figures 4(c) and 6(b)) [7, 10]. Similarly, deformation of mandibular dental arch, caused by lateral expansion did not return to normal (Figures 5(a)–5(c)) [9]. On the other hand, histopathological changes in the mandibular condyle (increases in cartilaginous layer width, bone matrix ratio, and number of osteoblasts) were reversible (Figures 7(c) and 8(c)–8(e)) [10]. These results suggest that the hypertrophic external form, modeled by endochondral ossification in the rat condyle, is stable. 

## 5. Conclusion

**Figure 9:**
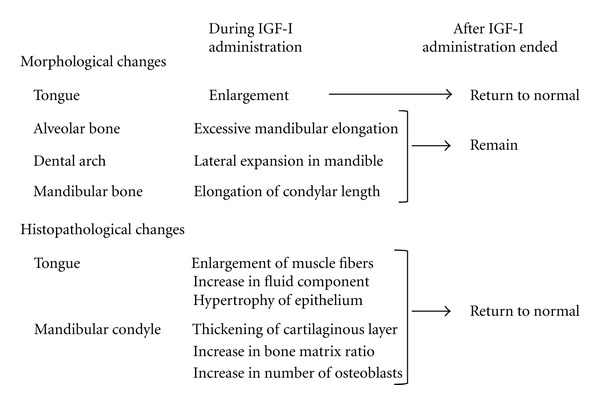
Changes in orofacial structures during and after IGF-I administration.

Figure 9 summarizes our studies on morphological and histopathological changes in orofacial structures of experimentally developed acromegaly-like rats. Exogenous administration of IGF-I evoked acromegaly-like specific enlargement of the tongue and mandible in rat. After normalization of the circulating IGF-I level, enlargement of the tongue and histopathological changes in the tongue and mandibular condyle also returned to normal, while skeletal morphological changes in the mandible remained. Taken together, these results suggest that the most suitable time to begin occlusal treatment in acromegalic patients is after improvement of tongue enlargement, because these treatments must be used as part of a comprehensive strategy to achieve functional harmony with the tongue. It is also clear that informed consent should be sought for additional surgical orthodontic treatment, because the enlarged mandibular bone may not return to a normal size on its own accord.

## References

[1] Ardran GM, Kemp FH (1972). The tongue and mouth in acromegaly. *Clinical Radiology*.

[2] Kunzler A, Farmand M (1991). Typical changes in the viscerocranium in acromegaly. *Journal of Cranio-Maxillo-Facial Surgery*.

[3] Takakura M, Kuroda T (1998). Morphologic analysis of dentofacial structure in patients with acromegaly. *International Journal of Adult Orthodontics and Orthognathic Surgery*.

[4] Dostálová S, Šonka K, Šmahel Z, Weiss V, Marek J (2003). Cephalometric assessment of cranial abnormalities in patients with acromegaly. *Journal of Cranio-Maxillo-Facial Surgery*.

[5] Herrmann BL, Mortsch F, Berg C, Weischer T, Mohr C, Mann K (2011). Acromegaly: a cross-sectional analysis of the oral and maxillofacial pathologies. *Experimental and Clinical Endocrinology and Diabetes*.

[6] Ebner FH, Kürschner V, Dietz K, Bültmann E, Nägele T, Honegger J (2010). Craniometric changes in patients with acromegaly from a surgical perspective. *Neurosurgical Focus*.

[7] Iikubo M, Ikeda H, Kobayashi A (2004). Insulin-like growth factor-I stimulates acromegaly-like specific mandibular enlargement in rats. *Hormone and Metabolic Research*.

[8] Kobayashi A, Iikubo M, Kojima I, Ikeda H, Sakamoto M, Sasano T (2006). Morphological and histopathological changes in tongues of experimentally developed acromegaly-like rats. *Hormone and Metabolic Research*.

[9] Iikubo M, Kobayashi A, Kojima I, Ikeda H, Sakamoto M, Sasano T (2008). Excessive lateral dental arch expansion in experimentally developed acromegaly-like rats. *Archives of Oral Biology*.

[10] Kojima I, Likubo M, Kobayashi A, Ikeda H, Sakamoto M, Sasano T (2008). High serum levels of IGF-I contribute to promotion of endochondral ossification in mandibular condyle and cause its specific elongation in acromegaly-like rats. *Hormone and Metabolic Research*.

[11] Lee PDK, Baker BK, Liu F, Kwan EYW, Hintz RL (1996). A homologous radioimmunoassay for rat insulin-like growth factor-I (IGF-I): implications for studies of human IGF-I physiology. *Journal of Clinical Endocrinology and Metabolism*.

[12] Bermann M, Jaffe CA, Tsai W, DeMott-Friberg R, Barkan AL (1994). Negative feedback regulation of pulsatile growth hormone secretion by insulin-like growth factor I. Involvement of hypothalamic somatostatin. *Journal of Clinical Investigation*.

[13] Wittmann AL (1977). Macroglossia in acromegaly and hypothyroidism. *Virchows Archiv*.

[14] Desnoyers L, Simonette RA, Vandlen RL, Fendly BM (2001). Novel non-isotopic method for the localization of receptors in tissue sections. *Journal of Histochemistry and Cytochemistry*.

[15] Donath MY, Gosteli-Peter MA, Hauri C, Froesch ER, Zapf J (1997). Insulin-like growth factor-I stimulates myofibrillar genes and modulates atrial natriuretic factor mRNA in rat heart. *European Journal of Endocrinology*.

[16] Ikkos D, Luft R, Sjogren B (1954). Body water and sodium in patients with acromegaly. *Journal of Clinical Investigation*.

[17] Palmieri G, Ikkos D (1965). Water and electrolyte content of muscle in acromegaly. *Acta Endocrinologica*.

[18] Bengtsson BA, Brummer RJ, Eden S, Bosaeus I, Lindstedt G (1989). Body composition in acromegaly: the effect of treatment. *Clinical Endocrinology*.

[19] Landin K, Petruson B, Jakobsson KE, Bengtsson BA (1993). Skeletal muscle sodium and potassium changes after successful surgery in acromegaly: relation to body composition, blood glucose, plasma insulin and blood pressure. *Acta Endocrinologica*.

[20] Dorup I, Clausen T (1995). Insulin-like growth factor I stimulates active Na^+^-K^+^ transport in rat soleus muscle. *American Journal of Physiology*.

[21] Hinds EC (1970). Noninflammatory bone disease. *Journal of Oral Surgery*.

[22] Malpe R, Baylink DJ, Linkhart TA, Wergedal JE, Mohan S (1997). Insulin-like growth factor (IGF)-I, -II, IGF binding proteins (IGFBP)-3, -4, and -5 levels in the conditioned media of normal human bone cells are skeletal site-dependent. *Journal of Bone and Mineral Research*.

[23] Chalk WO (1856). Partial dislocation of the lower jaw from an enlarged tongue. *Transaction of the Pathological Society*.

[24] Benda C (1902). Akromegalie. *Deutsche Klinik am Eingange Des Zwanzigsten Jahrhunderts*.

[25] Markovic M, Triscovic D (1978). Some results of occlusal and metric analysis of acromegalic cases. *International Journal of Orthodontics*.

[26] Schoenle E, Zapf J, Humbel RE, Froesch ER (1982). Insulin-like growth factor I stimulates growth in hypophysectomized rats. *Nature*.

[27] Isgaard J (1992). Expression and regulation of IGF-I in cartilage and skeletal muscle. *Growth Regulation*.

[28] Hunziker EB, Wagner J, Zapf J (1994). Differential effects of insulin-like growth factor I and growth hormone on developmental stages of rat growth plate chondrocytes in vivo. *Journal of Clinical Investigation*.

[29] Enlow DH, Enlow DH, Hans MG (1996). Growth of the mandible. *Essentials of Facial Growth*.

[30] Sarnat BG (1966). Developmental facial abnormalities and the temporomandibular joint. *Dental Clinics of North America*.

[31] Luder HU (1994). Perichondrial and endochondral components of mandibular condylar growth: morphometric and autoradiographic quantitation in rats. *Journal of Anatomy*.

[32] Blumenfeld I, Gaspar R, Laufer D, Livne E (2000). Enhancement of toluidine blue staining by transforming growth factor-*β*, insulin-like growth factor and growth hormone in the temporomandibular joint of aged mice. *Cells Tissues Organs*.

[33] Suzuki S, Itoh K, Ohyama K (2004). Local administration of IGF-I stimulates the growth of mandibular condyle in mature rats. *Journal of Orthodontics*.

[34] Visnapuu V, Peltomäki T, Rönning O, Vahlberg T, Helenius H (2001). Growth hormone and insulin-like growth factor I receptors in the temporomandibular joint of the rat. *Journal of Dental Research*.

[35] Delatte M, von den Hoff JW, Maltha JC, Kuijpers-Jagtman AM (2004). Growth stimulation of mandibular condyles and femoral heads of newborn rats by IGF-I. *Archives of Oral Biology*.

[36] Mueller K, Cortesi R, Modrowski D, Marie PJ (1994). Stimulation of trabecular bone formation by insulin-like growth factor I in adult ovariectomized rats. *American Journal of Physiology*.

[37] Wakisaka A, Tanaka H, Barnes J, Liang CT (1998). Effect of locally infused IGF-I on femoral gene expression and bone turnover activity in old rats. *Journal of Bone and Mineral Research*.

[38] Spencer EM, Liu CC, Si ECC, Howard GA (1991). In vivo actions of insulin-like growth factor-I (IGF-I) on bone formation and resorption in rats. *Bone*.

[39] Hill PA, Reynolds JJ, Meikle MC (1995). Osteoblasts mediate insulin-like growth factor-I and -II stimulation of osteoclast formation and function. *Endocrinology*.

[40] Ikeda H, Jokura H, Yoshimoto T (2001). Transsphenoidal surgery and adjuvant gamma knife treatment for growth hormone-secreting pituitary adenoma. *Journal of Neurosurgery*.

[41] Zhao G, Monier-Faugere MC, Langub MC (2000). Targeted overexpression of insulin-like growth factor I to osteoblasts of transgenic mice: increased trabecular bone volume without increased osteoblast proliferation. *Endocrinology*.

[42] Miquet JG, Giani JF, Martinez CS (2011). Prolonged exposure to GH impairs insulin signaling in the heart. *Journal of Molecular Endocrinology*.

[43] Tominaga A, Arita K, Kurisu K (1998). Effects of successful adenomectomy on body composition in acromegaly. *Endocrine Journal*.

[44] Brummer RJM, Lonn L, Kvist H, Grangard U, Bengtsson BA, Sjostrom L (1993). Adipose tissue and muscle volume determination by computed tomography in acromegaly, before and 1 year after adenomectomy. *European Journal of Clinical Investigation*.

